# The *Mycobacterium marinum mel2 *locus displays similarity to bacterial bioluminescence systems and plays a role in defense against reactive oxygen and nitrogen species

**DOI:** 10.1186/1471-2180-7-4

**Published:** 2007-01-19

**Authors:** Selvakumar Subbian, Parmod K Mehta, Suat LG Cirillo, Jeffrey D Cirillo

**Affiliations:** 1 Dept. Microbial and Molecular Pathogenesis, Texas A&M University Health Sciences Center – College of Medicine, College Station, TX 77843-1114, USA

## Abstract

**Background:**

Mycobacteria have developed a number of pathways that provide partial protection against both reactive oxygen species (ROS) and reactive nitrogen species (RNS). We recently identified a locus in *Mycobacterium marinum*, *mel2*, that plays a role during infection of macrophages. The molecular mechanism of *mel2 *action is not well understood.

**Results:**

To better understand the role of the *M. marinum mel2 *locus, we examined these genes for conserved motifs in silico. Striking similarities were observed between the *mel2 *locus and loci that encode bioluminescence in other bacterial species. Since bioluminescence systems can play a role in resistance to oxidative stress, we postulated that the *mel2 *locus might be important for mycobacterial resistance to ROS and RNS. We found that an *M. marinum *mutant in the first gene in this putative operon, *melF*, confers increased susceptibility to both ROS and RNS. This mutant is more susceptible to ROS and RNS together than either reactive species alone.

**Conclusion:**

These observations support a role for the *M. marinum mel2 *locus in resistance to oxidative stress and provide additional evidence that bioluminescence systems may have evolved from oxidative defense mechanisms.

## Background

Mycobacteria appear to have numerous molecular pathways responsible for their inherent resistance to reactive oxygen species (ROS) [1-3]. In most bacteria, oxidative stress induces a global regulator, OxyR, that induces detoxifying enzymes such as alkyl hydroperoxide reductase (AhpC) and catalase/hydroperoxidase I (KatG) [4,5]. During normal aerobic metabolism bacteria produce superoxide (O_2 _•^-^) that is converted to hydrogen peroxide (H_2_O_2_) and oxygen (O_2_) by superoxide dismutase and H_2_O_2 _is converted to water (H_2_O) and O_2 _by KatG [6] or AhpC [7]. The two superoxide dismutase (SOD) genes present in mycobacteria, *sodA *and *sodC*, have been suggested to play a role in resistance to ROS. A *sodC *mutant is more susceptible to ROS, including hydrogen peroxide (H_2_O_2_), and displays a defect in growth within activated macrophages [8,9]. The *sodA *gene has been down-regulated by antisense methods, resulting in increased sensitivity to H_2_O_2 _[10]. Mycobacteria also express a catalase, KatG, that affects resistance to ROS produced by NADPH oxidase activity in activated macrophages [2]. Other pathways must play an important role in resistance of *M. tuberculosis *to oxidative stress because *oxyR *is inactive [11], *katG *is absent or mutated in numerous human clinical isolates [12-16] and *ahpC *is expressed at very low levels [17,18].

Similar to ROS, there are several pathways involved in mycobacterial resistance to reactive nitrogen species (RNS), including *noxR1*, *noxR3 *[19,20], *dlaT *[21], *msrA *[22,23], *cysH *[3], DNA repair, protein degradation in the proteasome and flavin cofactor synthesis [24]. In addition to its role in resistance to ROS, the mycobacterial *ahpC *is also involved in resistance to the RNS peroxynitrite, but not nitric oxide [25]. Peroxynitrite is produced by SOD in the presence of H_2_O_2 _and nitric oxide, linking these two important mechanisms of oxidative stress-mediated cell death [26]. This observation may help to explain the inherent resistance of *M. tuberculosis *to peroxynitrite as compared to less pathogenic mycobacteria [27].

Bioluminescence systems can protect cells against ROS [28-32] through a catalase-like reaction between the electron donating ROS and oxidized luciferase-bound flavin mononucleotide, producing water and light [33]. The similarity of luciferases to oxidases [34] suggests that bioluminescence systems could have evolved from oxygen defense mechanisms [35]. During genetic analysis of factors that affect macrophage infection, we identified the *M. marinum mel2 *locus, which displays similarity to *lux *genes involved in bioluminescence [36]. In the current study, more detailed analysis of the genes in the *mel2 *locus suggests functional similarity between *mel2 *and bioluminescence systems. Based on this similarity, we asked whether the *M. marinum mel2 *locus is involved in resistance of mycobacteria to oxidative stress. We constructed an *M. marinum *mutant that carries a transposon insertion in the first gene in the *mel2 *locus, *melF*, by allelic exchange and demonstrated that this mutant displays increased susceptibility to both ROS and RNS. Since this mutation may have polar effects on downstream genes, we complemented this mutant with two constructs, one that carries the *melF *gene alone and another with the entire *mel2 *locus. The *melF *mutant defect is partially complemented by *melF *alone, but fully complemented by the entire *mel2 *locus. We recently found that the *mel2 *mutant displays a defect for growth in activated macrophages that is alleviated by the presence of either ROS scavengers or nitric oxide synthase inhibitors [37], suggesting that the *mel2 *mutant is more susceptible to ROS and RNS than wild type bacteria. The data obtained in the current study support and extend these observations through demonstration that the *mel2 *locus plays a role in susceptibility to several different compounds that produce ROS and RNS in laboratory media. Our results indicate that the *M. marinum mel2 *locus is the first of a newly identified class of genes with similarity to bioluminescence genes involved in resistance to both ROS and RNS.

## Results

### Similarity of the genes in the *mel2 *locus to bioluminescence genes

**Figure 1:**
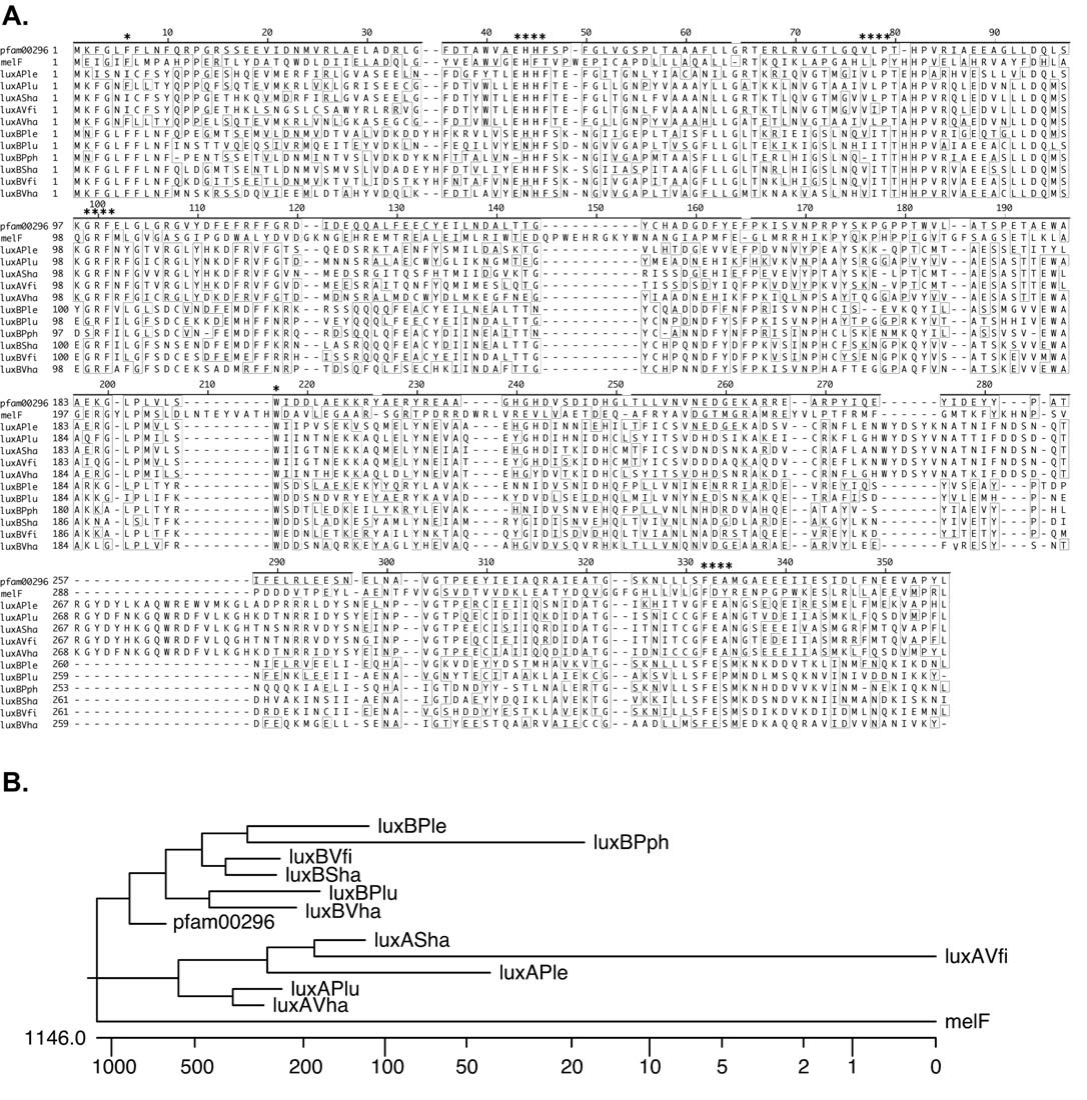
**Similarity of MelF to the bioluminescence protein luciferase**. Alignment of MelF with LuxA and LuxB proteins from bioluminescent bacteria and the concensus (pfam00296) (A) and a dendogram (B) showing the relationship between them. Numbers to the left of the alignment indicate the position within each protein and above indicate the position within pfam00296. Residues that match the concensus are shown outlined by boxes. Asterixes above alignment indicate conserved regions thought to be involved in enzyme activity. Abbreviations for aligned proteins and their accession numbers are as follows: melF, *M. marinum *MelF (AAV32084); pfam00296, luciferase-like monooxygenases conserved domain; luxAPle, *Photobacterium leiognathi *LuxA (P29238); luxAPlu, *Photorhabdus luminescens *LuxA (AAK98554); luxASha, *Shewanella hanedai *LuxA (BAB40796); luxAVfi, *Vibrio fishcheri *LuxA (AAD48477); luxAVha, *Vibrio harveyi *LuxA (CAA41597); luxBPle, *Photobacterium leiognathi *LuxB (P09141); luxBPlu, *Photorhabdus luminescens *LuxB (AAK98555); luxBPph, *Photobacterium phosphoreium *LuxB (P12744); luxBSha, *Shewanella hanedai *LuxB (BAB40797); luxBVfi, *Vibrio fishcheri *LuxB (AAD48478); luxBVha, *Vibrio harveyi *LuxB (AAA88686). The dendogram was rendered from the alignment using MegAlign (DNASTAR). Tree length is shown to the left of the log scale ruler beneath the dendogram.

The initial analysis of the genes present in the *mel2 *locus indicated that the *melF*, *melG *and *melH *genes display similarity to *luxA *[38], *luxG *[39] and *luxH *[39] genes involved in bioluminescence [36]. In order to obtain a better understanding of these findings and explore the possibility of additional functional similarities, we conducted detailed analysis of the conserved motifs present within the *melF*-*melK *genes. We first conducted an NCBI Conserved Domain (CD) Search with MelF. We obtained a 100% alignment (E = 1 × 10^-28^) for the 323 amino acid (a.a.) bacterial luciferase-like monooxygenase motif (pfam00296.11; Figure 1A). This motif is conserved in all bacterial luciferase genes, including the *luxA *and *luxB *genes from *Vibrio harveyi*, for which crystal structures have been previously determined [40,41]. Many of the residues responsible for catalytic activity and FMNH_2 _binding for LuxA and LuxB are also present in MelF [42,43], suggesting that these proteins have related activities. Analysis of the relatedness of MelF to LuxA and LuxB places MelF on an independent branch (Figure 1B), indicating it is nearly equally related to both, with a slightly closer relationship to LuxA than LuxB.

**Figure 2:**
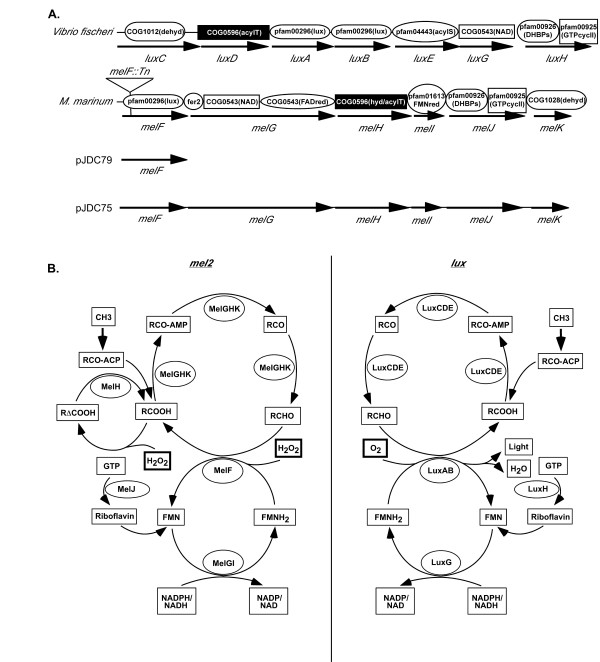
**Conserved motifs within the *mel2 *locus and our working model**. Conserved domains found within the *Vibrio fisheri lux *loci and *Mycobacterium marinum mel2 *locus (A) and proposed biochemical roles (B). Position of the *M. marinum mel2 *mutant transposon insertion is shown as a triangle above the *mel2 *locus (*melF::Tn*) and the structures of the complementing constructs, pJDC79 and pJDC75, are below the *mel2 *locus. The *luxC-luxG *region is located from the *V. fisheri *chromosome II and the *luxH *gene is from chromosome I (A). Conserved domains were identified using the NCBI conserved domain search. The designation for each conserved domain(s) is shown above each gene with an abbreviation for its proposed biochemical function in parenthesis. Abbreviations for proposed biochemical functions are as follows with additional conserved domains not shown in the figure in parenthesis: lux, luciferase; fer2, 2Fe-2S iron-sulfur cluster binding domain (cd00207); NAD, flavodoxin oxidoreductases and oxidoreductase NAD-binding domain (pfam00175); FADred, FAD-dependent oxidoreductases; hyd, abhydrolase alpha/beta hydrolase fold (pfam00561); acylT, predicted acyltransferases; FMNred, flavin reductase like domain; DHBPs, 3,4-dihydroxy-2-butanone 4-phosphate synthase; GTPcycII, GTP cyclohydrolase II; dehyd, dehydrogenase; acylS, acyl-protein synthetase. We constructed a hypothetical model for how these proteins might interact to reduce reactive oxygen species (B). The resulting pathways for *mel2 *are similar to the lux pathways, but are better adapted to serve as a potential defense against oxidative stress through the presence of an epoxide hydrolase (*melH*), in addition to the reduction of fatty acid aldehydes observed with lux. R represents a number of potential fatty acid molecules that could be used as substrates for these reactions. The LuxCDE proteins function as a complex to produce the aldehydes used to reduce oxygen by LuxAB and we have depicted a similar situation for MelGHK.

Analysis of conserved domains within melG-melK also demonstrated striking similarity to genes involved in bioluminescence (Figure 2A). Functional domains that display similarity to LuxC (MelK), LuxD (MelH), LuxG (MelG) and LuxH (MelJ) were identified. Although there is no clear homologue of LuxE within the *mel2 *locus, MelH carries domains with similarity to aminopeptidases and lysophospholipases, suggesting that this protein could serve in the role of both the transferase and synthetase activities found in the *lux *pathway. Additional putative functional domains were present within MelG, MelH and MelI that were not present within the *lux *genes. Some of these differences may be due to differences in substrate specificity between these pathways and may help to explain why mycobacteria are not luminescent. With the differences and similarities between the *mel2 *and *lux *proteins in mind, we constructed a working model for the putative biochemical roles of the proteins encoded by the *mel2 *locus (Figure 2B). Since mycobacteria face significant ROS during infections, we reasoned that a role in protection against ROS could help to explain the presence of conserved domains between the *mel2 *locus and bioluminescent systems.

### *M. marinum mel2 *mutant and complementing strains

**Figure 3:**
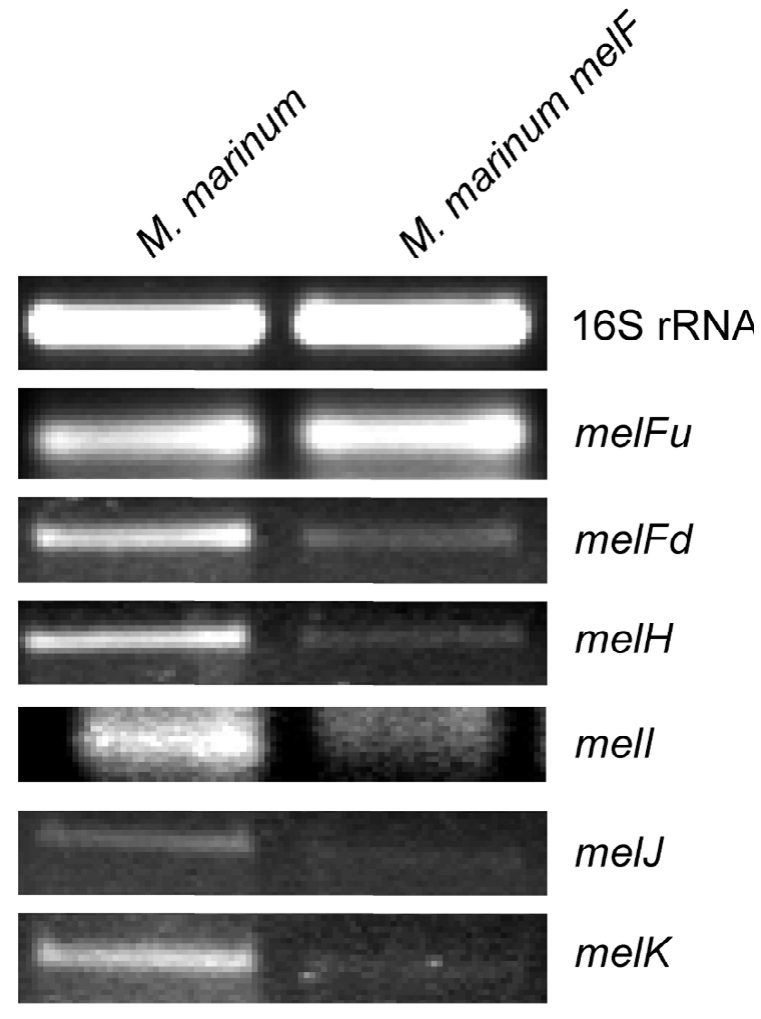
**Examination of the polar effects due to the *melF *insertion mutation**. RT-PCR studies to evaluate the effects of the Mu insertion on *mel2 *transcription upstream (*melFu*) and downstream of the insertion site (*melFd*, *melH*, *melI*, *melJ*, *melK*). Equivalent amounts of RNA from *M. marinum *wild type and the *M. marinum melF *mutant were reverse transcribed and subjected to PCR with specific oligonucleotides within each gene. Equal amounts of each PCR product were loaded on 0.8% agarose gels and compared to the 16S rRNA control RT-PCR reaction for each strain (16S rRNA) carried out on the same samples. Data shown are representative of two independent experiments.

An *M. marinum mel2 *mutant was constructed by in vitro mutagenesis of the *mel2 *locus with mini-*Mu *and replacement of the wild type gene by allelic exchange [36]. We confirmed the presence of the appropriate insertion in the *melF *gene (Figure 2A) by Southern analysis and PCR. Since insertion mutations can have polar effects on downstream genes and the genes in the *mel2 *locus are very closely juxtaposed to each other, we asked whether the insertion in *melF *affects transcript levels for the *melG*-*melK *genes. RT-PCR with primer pairs upstream of the *melF *insertion mutation produces relatively similar levels of product for *the M. marinum melF *mutant and wild type strains, but RT-PCR with primer pairs within the downstream genes produce less product in the *melF *mutant than the wild type strain (Figure 3). These observations suggest that the *Mu *insertion in *melF *has polar effects on downstream genes and full complementation of this mutation will most likely require the entire *mel2 *locus. Since the *luxA *gene plays a pivotal role in bioluminescence and similar genes, including *melF*, are thought to be oxidoreductases, it is possible that only the *melF *gene will be required for the role of *mel2 *in resistance to ROS. In order to differentiate between these possibilities we complemented the *M. marinum melF *mutant with both *melF *alone (pJDC79) and the entire *mel2 *locus (pJDC75) (Figure 2).

### The mycobacterial *mel2 *locus affects susceptibility to ROS

**Figure 4:**
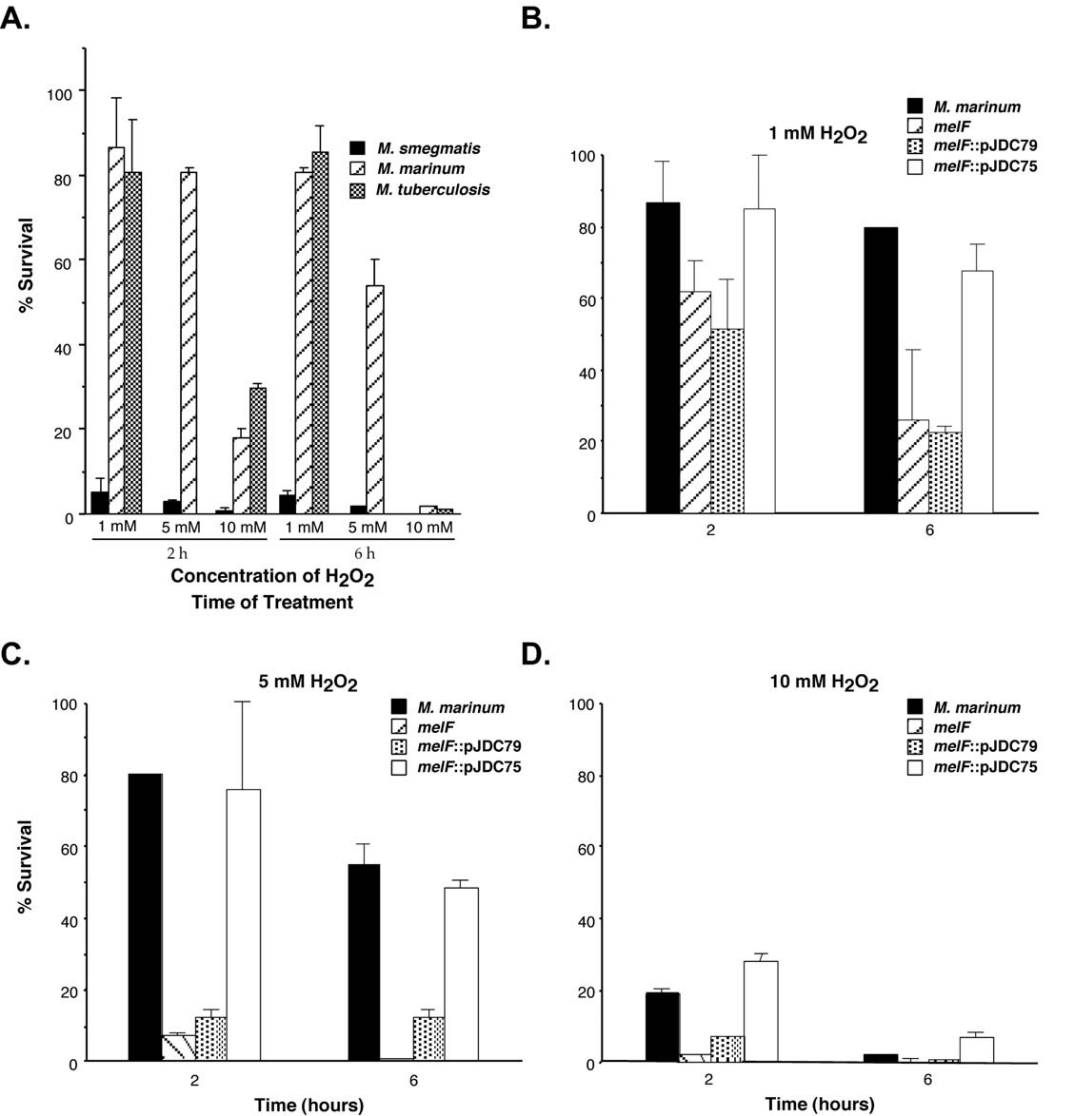
**Susceptibility of mycobacterial species and the *mel2 *mutant to H_2_O_2_**. Susceptibility of *M. smegmatis*, *M. marinum *and *M. tuberculosis *to different concentrations of H_2_O_2 _for 2 or 6 h (A). The data points are absent for *M. tuberculosis *at 5 mM H_2_O_2 _because these bacteria were only tested at 1 and 10 mM concentrations. Susceptibility of *M. marinum *wild type as compared to the *mel2 *mutant that carries an insertion in *melF *(*melF*), the mutant carrying a plasmid expressing *melF *alone (*melF*::pJDC79) and an integrated single copy plasmid expressing the entire *mel2 *locus (*melF*::pJDC75) to 1 mM (B), 5 mM (C) and 10 mM (D) H_2_O_2_. The % survival = (CFU Tx/CFU initial) × 100. Data are the means and standard deviations of assays done in triplicate, representative of three experiments.

We first compared the ROS susceptibility of wild type *M. marinum *with that of *M. tuberculosis *and the non-pathogenic mycobacterial species *M. smegmatis *(Figure 4A). We found that *M. marinum *displays similar levels of resistance to H_2_O_2 _as *M. tuberculosis *at various concentrations and times of treatment. In contrast, *M. smegmatis *is readily killed, even at 1 mM H_2_O_2 _where the pathogenic strains are nearly completely resistant (P < 0.001). Interestingly, we found that a *mel2 *mutant that carries an insertion in the *melF *gene is much more susceptible than wild type *M. marinum *to H_2_O_2 _(Figure 4B–D; P < 0.01). This difference is more pronounced at 5 mM (between 76–84% for wild type vs. 4–10% survival for the mutant after 2 h) than at 1 mM (between 85–97% for wild type vs. 52–70% survival for the mutant after 2 h). Resistance to H_2_O_2 _cannot be restored to the *mel2 *mutant with the *melF *gene alone, even expressed from a plasmid (pJDC79), but can be restored by a single copy integrated plasmid carrying the entire *mel2 *locus (pJDC75). In contrast, no difference in the growth rate or survival of these mycobacterial strains in standard laboratory medium without H_2_O_2 _is observed (data not shown). These observations suggests that the *melF *gene alone is not sufficient to confer resistance to H_2_O_2_, and that the *melF *insertion mutation has polar effects on downstream genes involved in H_2_O_2 _resistance.

**Figure 5:**
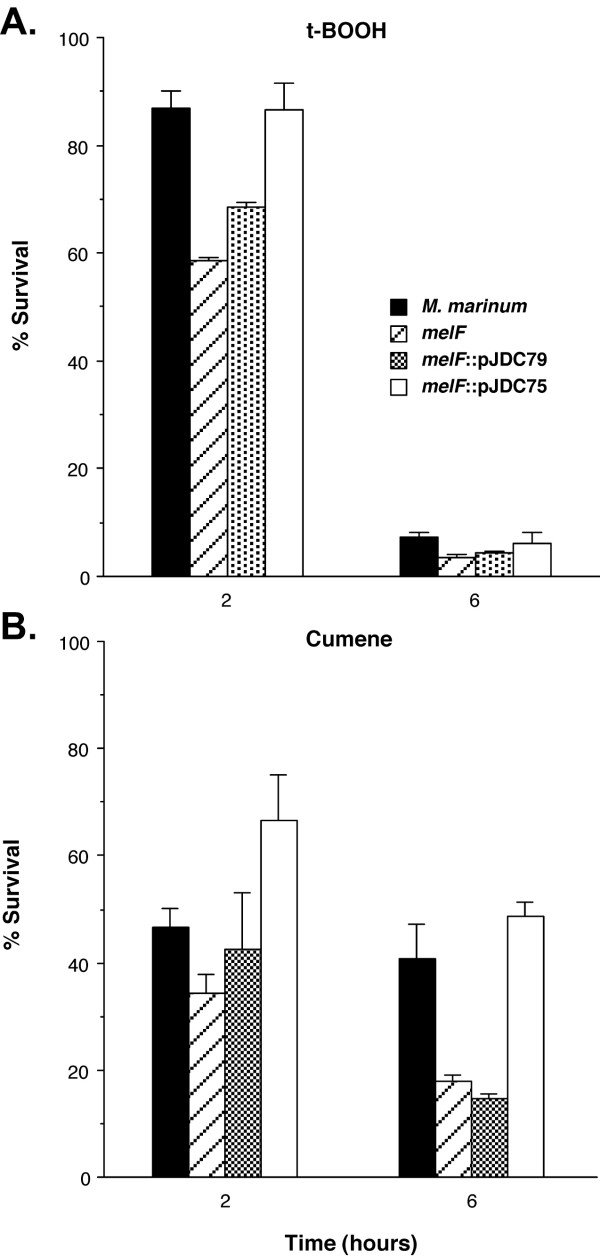
**Susceptibility of the *mel2 *mutant to reactive oxygen species**. Susceptibility of *M. marinum *wild type as compared to the *mel2 *mutant that carries an insertion in *melF *(*melF*), the mutant carrying a plasmid expressing *melF *alone (*melF*::pJDC79) and an integrated single copy plasmid expressing the entire *mel2 *locus (*melF*::pJDC75) to 10 mM tert-butyl hydroperoxide (t-BOOH, A) or 100 mM cumene hydroperoxide (Cumene, B). The % survival = (CFU Tx/CFU initial) × 100. Data are the means and standard deviations of assays done in triplicate, representative of three experiments.

We further probed the role of the *mel2 *locus in resistance to ROS through the use of two additional ROS generating compounds, cumene hydroperoxide and t-BOOH. Both of these compounds are organic peroxides that produce ROS inside the bacterial cell, but are more stable in aqueous solutions than H_2_O_2_. Organic peroxides decompose to alkoxyl and peroxyl radicals in addition to H_2_O_2 _[44,45]. The *mel2 *mutant was more susceptible to both cumene hydroperoxide and t-BOOH than wild type *M. marinum *(Figure 5; P < 0.01). Interestingly, at the two-hour time point partial complementation of the resistance defect was observed, but once again, the entire *mel2 *locus confers wild type resistance levels. These observations indicate that the *mel2 *locus plays a role in resistance to ROS, including the diverse radicals produced by organic peroxides.

### The *mel2 *locus affects susceptibility to RNS

**Figure 6:**
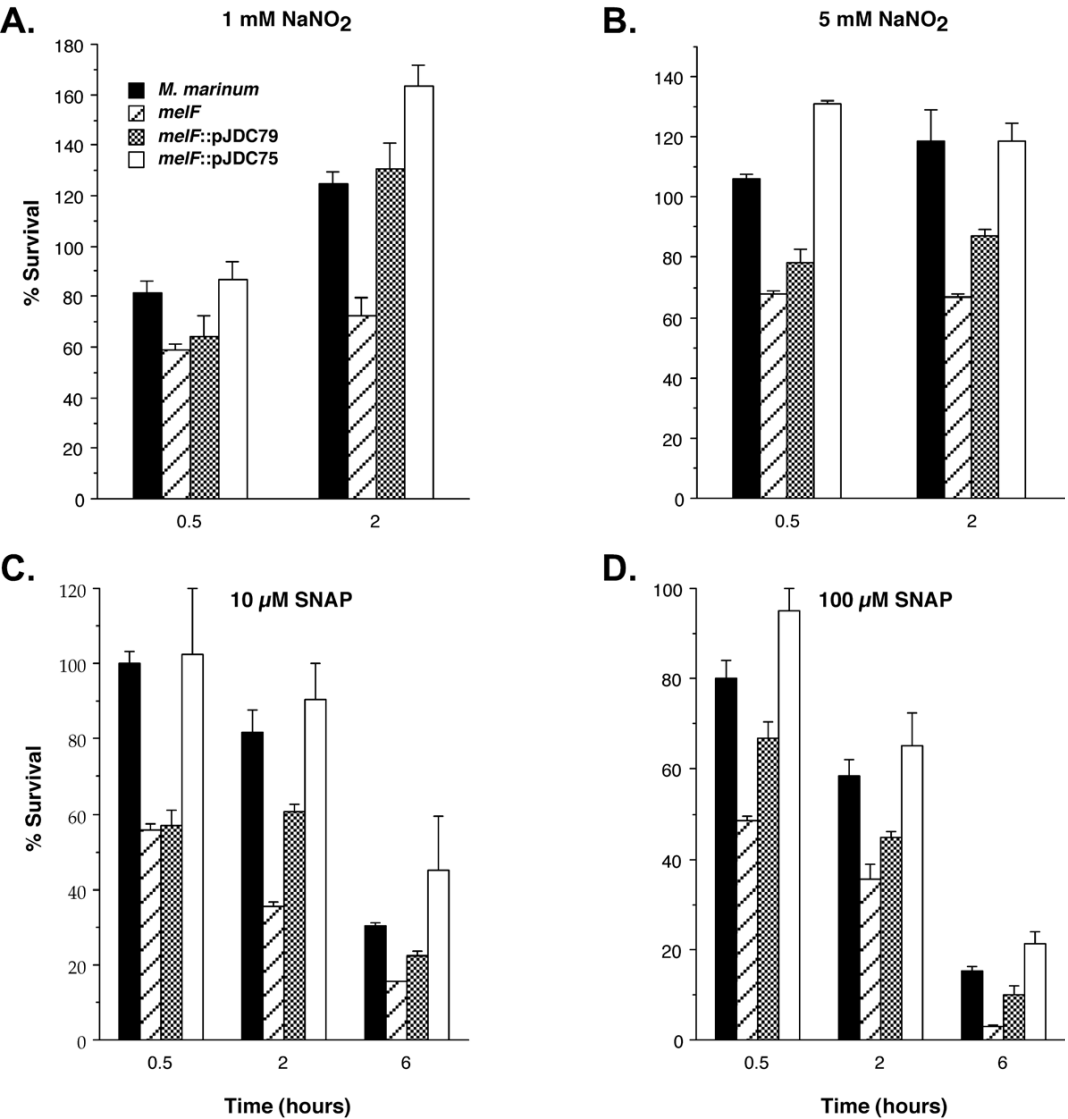
**Susceptibility of the *mel2 *mutant to reactive nitrogen species**. Susceptibility of *M. marinum *wild type as compared to the *mel2 *mutant that carries an insertion in *melF *(*melF*), the mutant carrying a plasmid expressing *melF *alone (*melF*::pJDC79) and an integrated single copy plasmid expressing the entire *mel2 *locus (*melF*::pJDC75) to 1 mM (A) or 5 mM (B) sodium nitrite (NaNO_2_) and 10 μM (C) or 100 μM (D) S-nitroso-N-acetyl penicillamine (SNAP). The % survival = (CFU Tx/CFU initial) × 100. Data are the means and standard deviations of assays done in triplicate, representative of three experiments.

Since the *mel2 *locus plays a role in resistance to ROS, it is also possible that it will affect resistance to RNS. The ROS and RNS pathways are linked in the reaction of nitric oxide with superoxide to produce peroxynitrite [1,46,47]. Because of the importance of RNS in protection against mycobacterial infections [48-51], pathways that affect susceptibility are likely to be important for pathogenesis. We examined the susceptibility of the *mel2 *mutant to acidified NaNO_2_, which is a source of nitric oxide [24,48], and SNAP, which releases nitric oxide under neutral pH in the presence of trace metals [52,53]. Similar to ROS, the *mel2 *mutant displays greater susceptibility than wild type *M. marinum *to RNS (P < 0.01) and this phenotype can be complemented partially by the *melF *gene alone and completely by the entire *mel2 *locus (Figure 6).

### The *mel2 *locus affects susceptibility to the combination of ROS and RNS

**Figure 7:**
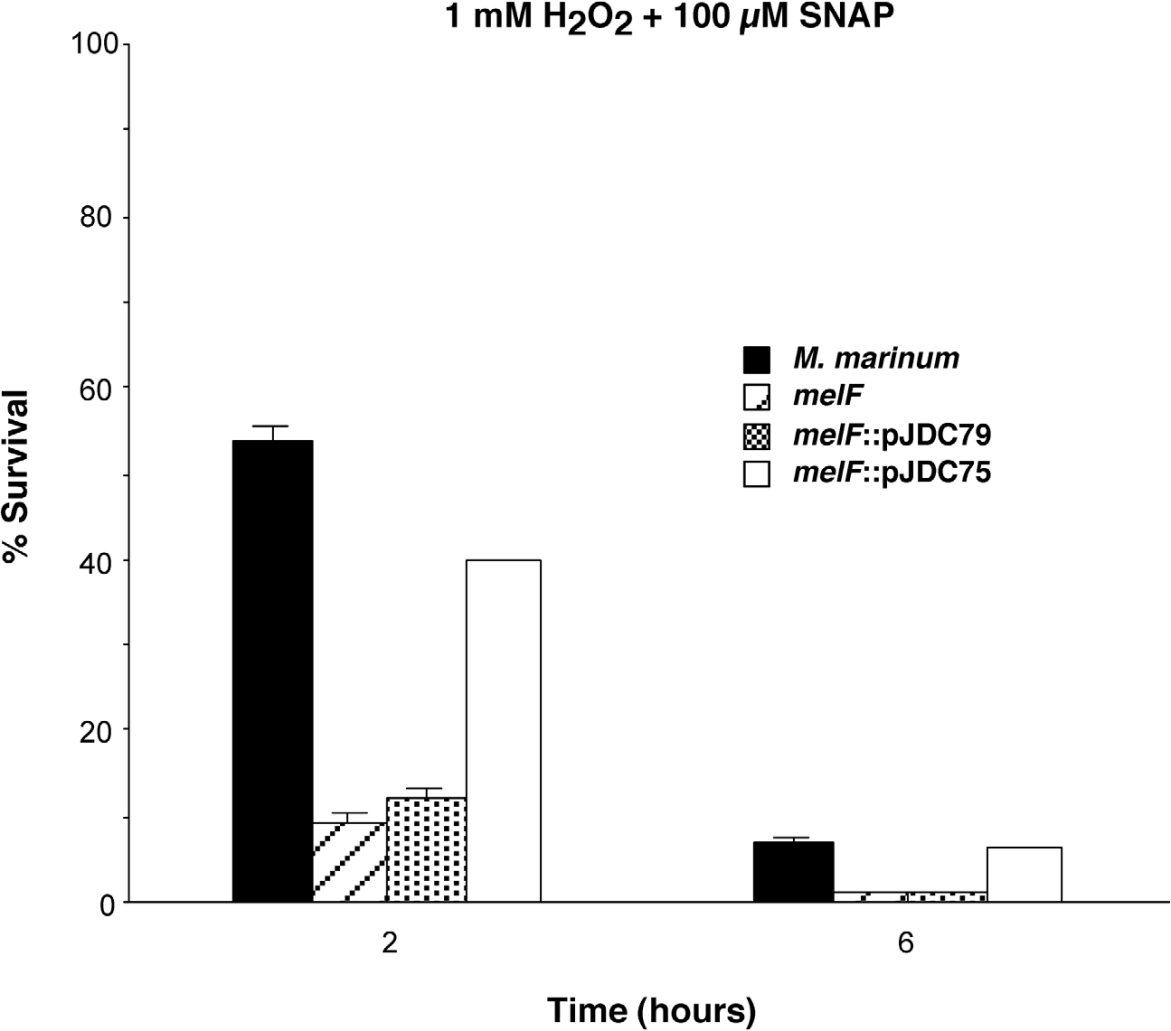
**Susceptibility to reactive nitrogen and oxygen species together**. Susceptibility of *M. marinum *wild type as compared to the *mel2 *mutant that carries an insertion in *melF *(*melF*), the mutant carrying a plasmid expressing *melF *alone (*melF*::pJDC79) and an integrated single copy plasmid expressing the entire *mel2 *locus (*melF*::pJDC75) to H_2_O_2 _plus S-nitroso-N-acetyl penicillamine (SNAP). The % survival = (CFU Tx/CFU initial) × 100. Data are the means and standard deviations of assays done in triplicate, representative of three experiments.

Since these observations suggest that the *mel2 *locus is involved in resistance of mycobacteria to both ROS and RNS, we examined whether the presence of both ROS and RNS simultaneously would have a more dramatic effect upon this mutant. Interestingly, the *mel2 *mutant is much more susceptible to treatment with both H_2_O_2 _and SNAP together than either compound alone (Figure 7; P < 0.01). These observations suggest that the *mel2 *mutant plays a role in susceptibility to both ROS and RNS, whether treated with them together, as most likely occurs in vivo, or separately.

## Discussion

The molecular mechanisms of mycobacterial resistance to ROS and RNS have been an area of intense investigation and suggest that there are multiple pathways involved in resistance [1]. In the current study, we identified a novel set of genes in the *mel2 *locus that play a role in resistance to both ROS and RNS. As shown in our previous studies, this locus is also important for survival in activated macrophages and virulence in the mouse footpad model of infection [37]. To the best of our knowledge, this is the first description of a mycobacterial pathway that impacts susceptibility to both of these reactive species. Since RNS and ROS are linked through the production of peroxynitrite from nitric oxide and superoxide [1,46,47], the *mel2 *system may be specifically involved in resistance to this reactive species. The presence of the *mel2 *locus in the tuberculosis complex and *M. marinum *[36] and absence in avirulent mycobacteria that are more susceptible to peroxynitrite [27] supports this concept.

The similarity of the *mel2 *locus to bioluminescence systems at the amino acid level and the presence of conserved domains between them are intriguing observations. These data are particularly interesting in light of the recent observations that bioluminescent systems can protect cells against oxidative stress [28-32]. In search of a biological role for bioluminescence in bacteria that would explain how such an energy-consuming system could have developed evolutionarily, it has been proposed that these pathways protect against ROS generated in an aerobic atmosphere [35,54]. Interestingly, it has been observed that ROS play a pivotal role in host-symbiont interactions with bioluminescent bacteria [55]. At present, our model for the biochemical function of *mel2 *(Figure 2B) is purely hypothetical and is in need of more experimental support, but the large number of conserved functional domain similarities between the *lux *and *mel2 *loci suggests that they may have related functions. However, it seems unlikely that this function is bioluminescence, since mycobacteria are not normally bioluminescent and we did not observe any bioluminescence associated with our mutant or complemented strains (data not shown). Our observation that the *mel2 *locus plays a role in resistance to ROS helps to explain the presence of loci similar to bioluminescence genes in non-luminescent bacterial pathogens.

The inherent resistance of *M. marinum *to ROS is impacted by a mutation in the *mel2 *locus. This observation suggests that *mel2 *has an important role in either directly scavenging oxygen radicals or repairing damage caused by them. Since the *mel2 *mutant affects susceptibility to H_2_O_2 _and the organic peroxides cumene hydroperoxide and t-BOOH, which generate alkoxyl radicals, peroxyl radicals and H_2_O_2 _[44,45], it is unclear whether *mel2 *is specific to a particular type of ROS. The apparent absence of specificity could be the result of this pathway utilizing an unknown oxidizable substrate that is recycled, similar to luciferin in bioluminescent systems [35], direct scavenging of H_2_O_2_, which all three compounds produce, or repair of damaged DNA, proteins or lipids [6]. Interestingly, luciferase can produce light using H_2_O_2 _alone, in the absence of luciferin, suggesting that luciferase can scavenge H_2_O_2_, superoxide and hydroxyl radicals [33]. Overall, these data suggest that MelF functions as a FMN-dependent non-heme catalase. The presence of the *mel2 *locus in pathogenic mycobacteria may at least partially explain why the catalase (*katG*) gene can be mutated during acquisition of isoniazid resistance [56], yet *katG *negative *M. tuberculosis *are responsible for numerous clinical infections in humans [12-16]. Since oxidative stress increases susceptibility of mycobacteria to isoniazid [57], it is possible that in some cases there is a relationship between isoniazid susceptibility and the *mel2 *locus. This possibility can be tested by comparing the effects of a double and single *katG *and *mel2 *mutants on virulence and isoniazid resistance.

The role of bioluminescence systems from other bacteria in resistance to RNS has not been examined, but our observations with *mel2 *suggest that this possibility is worth investigating. Since susceptibility to both SNAP and acidified NaNO_2 _are impacted by the *mel2 *mutation, this phenotype is not the result of greater susceptibility to the acidic pH used with NaNO_2_. The fact that the *mel2 *mutant displays an obvious defect when exposed to a combination of both ROS and RNS would imply that this locus is important for growth in environments where both of these reactive species are present, such as during infection of mammals. We found that the *M. marinum luxA *homologue, *melF*, may play an important role in resistance to both RNS and ROS, since this gene alone can partially complement what may be a polar mutation. Alternatively, this observation could be the result of low levels of expression of the remainder of genes within *mel2*, as a result of the polar mutation. This polar mutation would allow only low levels of the putative Mel2 protein complex to be formed and provide partial complementation once a functional *melF *gene is expressed. A better understanding of the biochemical roles of each of the *mel2 *genes and their importance in susceptibility to ROS and RNS will require analysis of each gene individually as well as in the presence or absence of each of the different Mel2 components.

## Conclusion

In this study, we confirmed that the *mel2 *locus plays a role in the susceptibility of *M. marinum *to ROS and RNS. Although this locus displays similarity to bioluminescent systems in other bacterial species, further biochemical studies are necessary to demonstrate the functional significance of the conserved domains that are present. These observations suggest that *mel2 *represents a previously unrecognized pathway for resistance of bacterial pathogens to ROS and RNS and support the concept that bioluminescence systems may have evolved from oxidative stress defense mechanisms.

## Methods

### Strains and growth conditions

*M. marinum *strain M, a clinical isolate obtained from the skin of a patient [58], was used in these studies. *M. marinum *strains were grown at 33°C in 7H9 broth (Difco, Detroit, Mich.) supplemented with 0.5% glycerol, 10% albumin-dextrose complex (ADC) and 0.25% Tween 80 (M-ADC-TW) for 5 days. *M. smegmatis *strain mc^2^155 [59] cultures were grown in M-ADC-TW for 3 days at 37°C and *M. tuberculosis *strain Erdman (ATCC35801) cultures were grown in M-ADC-TW for 10 days at 37°C. The number of viable bacteria was determined for each assay using the LIVE/DEAD assay (Molecular Probes, Eugene, OR.) and by plating dilutions for colony forming units (cfu) on 7H9 (M-ADC) agar (Difco, Detroit, Mich.). All inocula used were > 99% viable. *E. coli *strains were grown in Luria-Bertani (LB, Difco) media at 37°C. Where appropriate, kanamycin was added at a concentration of 25 μg/ml (*E. coli*) or 10 μg/ml (*M. marinum*).

### Construction of *M. marinum mel2 *mutant and complementing strains

The *M. marinum mel2 *mutant carries a mini-*Mu *transposon insertion near the amino terminus of the *melF *gene as described previously [36]. Our previous studies have found no functional differences between the *M. tuberculosis *and *M. marinum mel2 *loci, both confer wild type host cell infection and growth in macrophages to the *M. marinum melF *insertion mutant [36,37], so either can be used for complementation studies. The *M. marinum melF*::pJDC79 strain is the *melF *mutant that carries the plasmid pMV262 [60] expressing the *melF *gene from *M. tuberculosis *that has been previously shown to complement the macrophage infection defect of the *M. marinum mel2 *mutant [36]. The *M. marinum melF*::pJDC75 strain is the *melF *mutant that carries the single-copy integrating plasmid pYUB178 [61] with the entire *M. tuberculosis mel2 *locus cloned into its single NheI site. Construction of all strains was confirmed by Southern analyses and PCR as described previously [36].

### RT-PCR analyses

RT-PCR for the *mel2 *transcripts was performed using the ThermoScript RT-PCR System (Invitrogen) according to the manufacturer's instructions. Basically, 300–500 ng of DNase treated, total bacterial RNA was mixed with gene specific reverse primers, dNTP mix and 40U of RNaseOUT and incubated at 65°C for 5 min and then placed on ice prior to use. The annealed primers were extended with 15 U of Thermoscript RT at 55°C for 60 min followed by heat inactivation of the enzyme at 85°C for 5 min. The residual, non-transcribed RNA were removed with 2U of *E. coli *RNaseH at 37°C for 20 min. 2 μl of the cDNA was used in PCR amplification with 1 mM appropriate forward and reverse primers and 5U of Thermopol enzyme (NEB) in a total volume of 50 ul. All primers used for RT-PCR reactions are shown in Table 1. The concentrations of RNA in wild type and mutant strains were normalized against the respective 16s rRNA. 150 ng of *M. marinum *total genomic DNA of was used as positive control for the PCR reaction and RT-PCR reactions without reverse transcriptase was included in all experiments as negative control. The amplified products were analyzed by 0.8% agarose gel electrophoresis and the products measured by densitometry semiquantitatively using an Alpha Imager (Alpha Innotech) and Alpha Ease FC software.

### In silico analysis of the *melF-melK *genes

Detailed analysis of the amino acid sequence of MelF-MelK was carried out initially using protein-protein National Center for Biotechnology Information (NCBI) BLAST [62] and Conserved Domain Search [63] as described previously [36]. Once motifs of interest were identified, they were compared to the appropriate bioluminescence genes and the *mel2 *gene and homologues were aligned and dendograms constructed using MegAlign (DNASTAR). Domain scores were considered significant if greater than 150 and the expectation values were less than 1 × 10^-10^.

### Susceptibility to reactive oxygen species

Mycobacterial strains were exposed to ROS generated by H_2_O_2_, cumene hydroperoxide and tert-butyl hydroperoxide (t-BOOH). The susceptibility of mycobacteria to these compounds was determined by treatment for various periods of time at the appropriate growth temperature for the mycobacterial strain used and plating dilutions on M-ADC agar to determine CFU at each time point as compared to the original inoculum (To), i.e. percent survival = (CFU Tx/CFU To) × 100. Dimethyl sulfoxide (DMSO) was used as a solvent for t-BOOH and was tested for effects on viability of all mycobacterial strains and no solvent affected mycobacterial viability during the time periods examined or at the final concentrations used.

### Susceptibility to reactive nitrogen species

Mycobacterial strains were exposed to RNS generated by S-nitroso-N-acetyl penicillamine (SNAP) and acidification of sodium nitrite (NaNO_2_) to pH 5.2 for various periods of time. Susceptibility was determined in the same manner as that described for ROS. DMSO was used as a solvent for SNAP and had no effects on viability of mycobacteria at the concentrations and time periods used.

### Statistical analyses

All experiments were carried out in triplicate and repeated at least three times. The significance of the results was determined using the Student t-test. *P *values of < 0.05 were considered significant.

## Authors' contributions

S.S. carried out the majority of these studies, participated in data analysis and participated in preparation of the manuscript. P.K.M. carried out some of the assays and participated in data analysis. S.L.G.C. carried out some of the assays and participated in data analysis. J.D.C. conceived the study, designed the experiments, completed the data analysis and prepared the final draft of the manuscript. All authors read the manuscript, participated in editing the manuscript and approved the final version.

## Figures and Tables

**Table 1:** Oligonucleotides

Name^a^	Target^b^	Sequence (5' -> 3')
MelFuF	*melFu*	CAGAAGACGCGATCACGGCG
MelFuR		GGGTCGGCGAACACTTCACC
MelFdF	*melFd*	CCTGCTGCCCTATCATCACC
MelFdR		CCTCCAAAGCCCGAAGCCGC
MelFtnF	*melF*tn	CAGAAGACGCGATCACGGCG
MelFtnR		CCTGCTGCCCTATCATCACC
MelGF	*melG*	GCGAGAAGGGCACCGCCATG
MelGR		CTCAGATCACCCACGGTCAC
MelHF	*melH*	GTGACCGTGGGTGATCTGAG
MelHR		GAAGGCGCAACTCACTGCCG
MelIF	*melI*	CGGCAGTGAGTTGCGCCTTC
MelIR		CATCCAGGCTCCGTTGCGGG
MelJF	*melJ*	CCCGCAACGGAGCCTGGATG
MelJR		GCCGCGAGTGGCGTGTCTGC
MelKF	*melK*	GCAGACACGCCACTCGCGGC
MelKR		GACGCTCACCACAGTGCGGC
rRNAF	rRNA	AGAGTTTGATCCTGGCTCAG
rRNAR		CACGCTCACAGTTAAGCTGT

^a^Designation for each oligonucleotide used in this study for RT-PCR analyses. F indicates a forward primer and R indicates a reverse primer.

^b^Target gene mRNA transcript for RT-PCR analyses. Oligonucleotides are listed as primer pairs. The reverse primers were used for cDNA synthesis and the forward and reverse used together for RT-PCR. The *melFu *target is the region of the mel2 locus transcript upstream of the transposon insertion in the *melF *mutant but down stream of the start codon, *melFd *target is the region downstream of the transposon insertion but still within *melF *and *melFtn *is across the transposon insertion in *melF*.
